# A Systematic Review of Lifestyle Interventions for Neuropathy and Neuropathic Pain: Smoking Cessation

**DOI:** 10.3390/neurosci6030074

**Published:** 2025-08-07

**Authors:** Michael Klowak, Rachel Lau, Mariyam N. Mohammed, Afia Birago, Bethel Samson, Layla Ahmed, Camille Renee, Milca Meconnen, Ezra Bado, Aquilla Reid-John, Andrea K. Boggild

**Affiliations:** 1 Institute of Medical Science, University of Toronto, Toronto, ON M5S 1A8, Canada; 2 Public Health Ontario Laboratories, Public Health Ontario, Toronto, ON M5G 1M1, Canada; 3 Tropical Disease Unit, Toronto General Hospital, Toronto, ON M5G 2C4, Canada; 4 Department of Medicine, University of Toronto, Toronto, ON M5S 3H2, Canada

**Keywords:** lifestyle medicine, neuropathy, neuropathic pain, smoking, smoking cessation, avoiding risky substances, systematic review

## Abstract

**Background:** Neuropathic pain (NP), resulting from damage to the somatosensory nervous system, affects 7–10% of the global population and remains poorly managed despite available therapies. Smoking has been associated with increased pain severity and disease burden, yet its role in neuropathy/NP has not been systematically reviewed. This systematic review synthesizes the existing literature on smoking status and its relationship with neuropathy/NP incidence, prevalence, and severity. **Methods:** The review was conducted in accordance with PRISMA guidelines and included studies that assessed smoking consumption, dependency, quantity, and cessation in individuals with neuropathy/NP. Summary estimates were stratified by exposure type, and pooled odds ratios and relative risks were calculated. **Results:** Across 62 studies comprising cohort, case–control, and cross-sectional designs, smoking was consistently associated with greater NP prevalence and pain severity. Smoking dependency was linked to increased incidence, while cessation was associated with reduced risk of NP. Despite considerable heterogeneity and risk of bias, particularly from subjective exposure measurement and inconsistent classification, this relationship remained statistically significant. **Conclusions:** Findings support the role of smoking as a modifiable risk factor in various etiologies of neuropathy/NP. Cessation may represent a low-cost, low-risk, low-tech adjunctive therapy; however, further robust cessation interventional trials are needed, particularly for less common infectious causes of chronic NP such as leprosy.

## 1. Introduction

Recent estimates indicate that neuropathic pain (NP), defined as pain caused by a lesion or disease of the somatosensory nervous system, affects 7–10% of the global population [1]. Its incidence is expected to rise with the increasing prevalence of common underlying etiologies, such as diabetes, cancer, immunotherapy, trauma/surgery, and persistently prevalent infections such as leprosy [1]. NP also imposes a substantial economic and individual burden. Direct medical costs are up to three times higher in patients with NP vs. healthy controls (USD 17,355 vs. 5715), with productivity and lifetime earnings losses up to USD 89,000, and disability rates exceeding 40% in some cohorts [2]. Despite available pharmacological treatments, NP remains poorly managed, with only 30–40% of patients achieving meaningful relief, defined as a 50% reduction in overall severity [3]. Systematic reviews also indicate declining treatment efficacy of existing interventions, as reflected in rising number needed to treat (NNT) values. The NNT for tricyclic antidepressants (TCAs), serotonin–norepinephrine reuptake inhibitors (SNRIs), and gabapentinoids have risen from 2.1–2.8, 5.0, and 4.2–6.4 to 3.6, 12.51, and 8.4, respectively [4,5,6]. While this trend may partially reflect methodological limitations in newer trials and off-label/expanded usage, it underscores the ongoing burden of NP and shortcomings of current gold-standard therapies, highlighting the need for effective supplantive or adjunctive approaches [7]. Lifestyle interventions, such as diet modification and routine exercise, have recently emerged as low-cost, accessible strategies for alleviating NP across various etiologies. Beyond these proactive/protective approaches, risk-reducing strategies, including minimizing exposure to risky neuropathic substances (such as tobacco, alcohol, aerosolized insecticides, and other environmental toxins), may play a comparable role in managing NP severity and progression [8,9,10,11,12].

Previous research has explored the relationship between smoking status and pain more broadly, with studies estimating that 42–68% of individuals with chronic pain are current smokers, nearly twice the rate observed in healthy controls. Some studies have also reported greater pain severity in current smokers compared to former and non-smokers (*p* < 0.05), suggesting a possible dose–response relationship [13,14,15]. These findings have led to proposed mechanisms linking chronic smoking with altered pain processing, including structural damage, inflammation, and nervous system dysregulation. Smoking may disrupt pain modulation by desensitizing nicotine acetylcholine receptors, suppressing endogenous opioid release, altering hypothalamic–pituitary–adrenal axis function, and impairing gamma-aminobutyric acid (GABA) pathways [14,16,17,18,19,20]. This maladaptive neurophysiological response has been hypothesized to contribute to a cycle of heightened pain perception and nicotine dependence, which may worsen overall pain outcomes in some individuals and increase the burden of certain non-infectious conditions [14,16,17,18,19,20]. Beyond these common and frequently observed mechanisms, smoking has also been correlated with persistently prevalent yet underrepresented infectious diseases outcomes. In leprosy, smoking prevalence exceeds that of the general population, reaching up to 37% in some cohorts, and has been significantly associated with greater disease severity, including more extensive ulceration, and impaired wound healing [21,22,23]. While most of this evidence relates to chronic pain in general, these pathways may also have relevance in the context of neuropathic pain. However, infectious causes of neuropathy, including leprosy, remain notably underexplored in relation to modifiable lifestyle risk factors such as smoking—an area in which this systematic review series may offer a foundational contribution.

Given this association and beyond the numerous other health benefits, smoking cessation has emerged as a crucial component of pain management. While nicotine withdrawal may cause temporary hyperalgesia, long-term smoking cessation is associated with reduced pain sensitivity and severity (*p* < 0.001), ultimately resulting in enhanced therapeutic management of chronic pain [14,24]. Despite this evidence, the effects of smoking status on neuropathy and NP, across both non-infectious and often neglected infectious etiologies, have yet to be systematically reviewed in the literature. Consequently, as part of an ongoing systematic review series on lifestyle interventions for neuropathy/NP, this systematic review seeks to address this knowledge gap by synthesizing the existing literature. It is hypothesized that smoking status significantly influences the incidence, prevalence, and severity of neuropathy and NP, and that cessation may ultimately improve overall morbidity.

## 2. Materials and Methods

**Figure 1:**
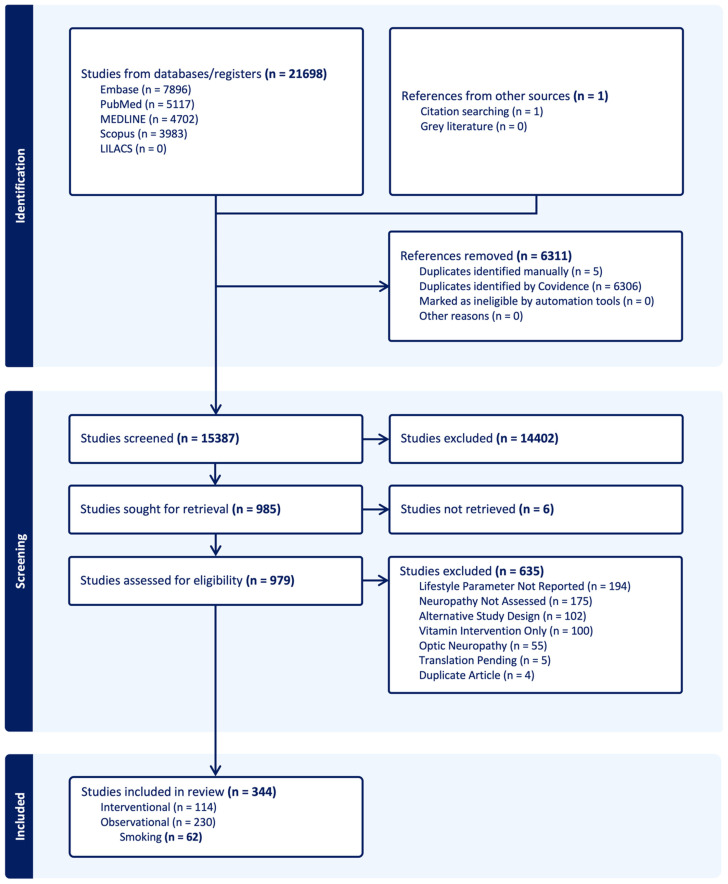
PRISMA flowchart.

This systematic review was conducted according to the “Preferred Reporting Items for Systematic Reviews and Meta-Analysis” (PRISMA) guidelines and is registered with the International Prospective Register of Systematic Reviews (PROSPERO: 484158) (Figure 1). It includes interventional and observational (cohort, case–control, and cross-sectional) studies that examined lifestyle factors such as interventions, exposures, outcomes, or stratification variables in individuals with neuropathy and/or NP of any cause. Studies were identified from inception to 6 August 2024. As part of an ongoing systematic review series on lifestyle interventions and neuropathy/neuropathic pain, this review focuses specifically on smoking-related exposures. Although the overarching search strategy included multiple lifestyle domains (i.e., diet, exercise, alcohol, smoking), only studies examining smoking consumption, dependency, or cessation were included in this particular review. Comprehensive methodological details of the systematic review series in general, including outcome measures, data sources, data extraction, and statistical analysis, have been previously published and can be found elsewhere [12].

### 2.1. Inclusion/Exclusion Criteria According to PICO Framework

Population: Individuals of any age or sex with neuropathy or neuropathic pain of any etiology, as identified and defined by the original paper [12].

Exposure: Smoking-related parameters, including consumption status (current/former/never), dependency (addiction or tobacco use disorder), quantity (i.e., pack-years), or cessation.

Comparator: Non-smokers or individuals with differing levels of smoking exposure.

Outcomes: Neuropathy or NP incidence, prevalence, and/or severity.

Study Design: Eligible studies included randomized controlled trials, cohort studies, case–control studies, cross-sectional studies, case series, and case reports (n ≥ 1). We excluded animal studies, trial protocols, conference abstracts, editorials, commentaries, and studies lacking relevant smoking or neuropathy-related outcomes. We also excluded articles focused on ocular neuropathies, given the lack of globally standardized reporting, the elevated risk of confounding from comorbid eye conditions like retinopathy or cataracts, and the distinct nature of non-ocular neuropathic mechanisms.

### 2.2. Search Strategy

An extensive search strategy was applied across five databases (Medline, PubMed, Scopus, Embase, and LILACS), incorporating underlying neuropathic conditions, relevant lifestyle factors, and key stratifying variables:

(neuropathic pain OR neuropathy OR neuritis OR diabetic neuropathy OR peripheral neuropathy OR chemical neuropathy OR toxic neuropathy OR chemotherapy-induced peripheral neuropathy OR vitamin B deficiency) AND (nutrition OR nutrient OR nutritionally compromised OR micronutrient OR macronutrient OR malnutrition OR nutritional status OR nutrient supplement* OR plant based OR vegetarian OR vegan OR mediterranean diet OR diet OR physical activity OR exercise OR lifestyle OR lifestyle interventions OR BMI OR smoking OR alcohol OR stress OR sleep).

### 2.3. Study Selection

All articles were screened for relevance, and studies were excluded if they did not explicitly address both lifestyle-related factors or interventions—in the case of this particular systematic review, smoking—and neuropathy/NP. Eligibility was determined based on the following inclusion criteria:

(i) Any patient population irrespective of age or sex;

(ii) Neuropathy/NP due to any cause (including non-specific neuropathy, neuritis, diabetic neuropathy, peripheral neuropathy, autonomic neuropathy, chemical neuropathy, toxic neuropathy, nutritional neuropathy, infectious neuropathy, and chemotherapy-induced peripheral neuropathy);

(iii) Treated with or assessed for a lifestyle intervention or parameter (including nutritional interventions, malnutrition, plant-based diets, vegetarian diets, vegan diets, Mediterranean diets, other types of diet, physical activity, exercise, body mass index (BMI), smoking, alcohol, stress, or sleep).

Document management, including deduplication, title/abstract screening, and full-text review, was conducted using Covidence. Two reviewers independently screened all records, with discrepancies resolved by a third reviewer acting as an arbitrator.

### 2.4. Risk of Bias and Certainty of Evidence

Risk of bias was assessed using customized forms based on the Joanna Briggs Institute critical appraisal tools. Methodological quality was evaluated using the GRADE framework, which rated each included study as high, moderate, low, or very low quality, depending on the observed risk of bias. The GRADE framework is a widely accepted and transparent method for evaluating certainty of evidence and the strength of recommendations. By default, randomized controlled trials receive a high-certainty rating, whereas observational studies are initially rated as low. These ratings may be downgraded due to various biases, such as selection, confounding, attrition, reporting, or information/outcome, or upgraded based on factors like strong effect sizes, dose–response relationships, or the presence of plausible confounders. Two reviewers independently and concurrently conducted these assessments, and any disagreements were resolved through discussion with a third reviewer serving as an arbitrator.

The methodology for identifying and assessing risk of bias and certainty of evidence has been previously reported for interventional trials [12]. In this systematic review, additional items specific to the observational studies under review are examined, as detailed. Selection and detection bias were identified based on whether study groups were defined using comparable criteria and appropriately matched, and whether uniform methods were applied in identifying cases/controls, respectively. Attrition bias was evaluated by examining participant loss. Losses exceeding 10% within a group, imbalances between groups due to withdrawals, or lack of reporting altogether were all considered potential sources of bias. Confounding bias was judged based on whether appropriate statistical adjustments were made, while information/outcome bias was noted if assessments were subjective by nature (such as self-reported questionnaires) or lacked objective measurements. Studies that failed to adequately account for these biases were deemed to carry moderate risk, whereas those that did not address these factors at all were considered high risk in their respective domains. Studies received a single overall risk-of-bias score based on pooled assessments of individual bias domains. Studies were rated as having an overall low risk of bias if the majority of the domains were low risk, with no more than one unclear domain. The presence of a single high-risk domain or multiple unclear-risk ratings resulted in a downgrade to an overall unclear risk of bias. Studies with multiple high-risk ratings or combinations of high and unclear risks were classified as having an overall high risk of bias.

Additional GRADE domains, such as inconsistency, indirectness, imprecision, effect size, and dose–response, were also evaluated when determining certainty of evidence. Studies were downgraded for inconsistency when there was substantial heterogeneity in findings, for imprecision when pooled confidence intervals were wide or crossed the null, and for indirectness when lifestyle factors were not the primary focus of the original analysis. Certainty was upgraded by one level for large effect sizes (<0.5 or >2), and by two levels for very large effects (<0.2 or >5). Final GRADE scores were assigned per outcome, integrating overall risk of bias with these considerations.

### 2.5. Meta-Analysis

Data were stratified by type of smoking exposure to categorize differences in reported consumption patterns and dependency. Stratification included consumption status (never, former, current), dependency measures (addiction and abuse/use disorders), quantity (on a yearly/daily basis), and smoking cessation (including quit attempts). However, variability in how these exposures were defined and measured across studies was anticipated, and exposure data were extracted at the highest level of detail provided by each study. Classification ranged from simple dichotomous variables (e.g., consumption yes/no) to more detailed categorizations (e.g., current vs. former vs. never), as well as broad quantity estimates (>100 cigarettes over a lifetime or >10 cigarettes per day). In some cases, broad classifications (e.g., “no” consumption vs. “current” consumption) may have included individuals with past exposure, leading to categorical inconsistencies. These sources of anticipated effect heterogeneity influence incidence and prevalence outcomes in this review by introducing variability in how exposure was classified and measured across studies. Regardless, exposure data were extracted at the highest level of detail reported by each study and summarized in Table 1, encompassing study characteristics.

Although studies specified neuropathy subtypes and etiologies, there was insufficient overlap across studies to allow for meaningful subgroup analysis. Due to variability in neuropathy classification across studies, all neuropathy outcomes were pooled. However, ambiguous or imprecise classifications (e.g., “moderate neuropathy” or “moderate sensory nerve action potential (SNAP) amplitudes”) were excluded, as were outcomes that could not be reliably translated into a categorical exposure yes/no classification.

To quantify these associations, odds ratios were used to reflect the association between smoking consumption and neuropathy, while relative risk compared the risk of developing neuropathy in the exposed (smoking) versus unexposed (non-smoking) groups. Summary estimates of both continuous and dichotomous outcomes were pooled for each combination of smoking exposure (consumption, dependency, quantity, and cessation) and neuropathy incidence and/or prevalence (Table 2). The significance level for all statistical tests was set at α = 0.05. Forest plots were generated for pooled odds ratios when multiple studies of the same design reported comparable smoking exposure classifications and neuropathy outcomes (GraphPad Prism v10.4.1, GraphPad, La Jolla, CA, USA). Pooled estimates were stratified across subgroups, including smoking consumption status, dependency, quantity, and cessation. All statistical analyses were conducted using PRISM v.10.4.1 (GraphPad, La Jolla, CA, USA).

## 3. Results

### 3.1. Literature Search

A total of 21,698 records were retrieved from five databases: Embase (7896), PubMed (5117), Medline (4702), Scopus (3983), and LILACS (0). An additional record was identified through bibliography screening. After removing duplicates, 15,387 unique records remained, with 985 full-text articles undergoing evaluation for final inclusion. Of these, 344 studies reported primary outcomes relevant to the overall systematic review on lifestyle factors and neuropathy/NP, including 62 that specifically examined smoking in relation to neuropathy or neuropathic pain (Table 1). A detailed account of the screening process, including the number of exclusions and reasons for study omission, is provided in Figure 1.

**Table 1:** Characteristics of all observational studies of smoking and neuropathy/NP included in this systematic review.

Author (Year)	Study Design	Setting	N	Sex N (F:M)	Age (Mean ± SD (Range))	Population/Etiology	Lifestyle	Outcomes
**Adler (1997) [25]**	Cohort Study	US	With Ne: 58; Without Ne: 230	With Ne: 1:57; Without Ne: 11:219	With Ne: 64.0; Without Ne: 61.5	DM ± Incident Ne	Smoking: ever, current	Current smoking was significantly associated with lower odds of incident Ne (10.3% vs. 26.1%, *p* = 0.011; β = −1.52, SE = 0.5635, 0.22 [0.07–0.66], *p* = 0.007). Authors suggest individuals with Ne may quit smoking due to emerging impairment (reverse causation—protopathic bias).
**Braffett (2020) [26]**	Cohort Study	US	With DPN: 455; Without DPN: 931	With DPN: 182:273; Without DPN: 475:456	^ With DPN: 29 (24, 34); Without DPN 26 (21, 32)	T1DM ± DPN	Cigarette Smoker	Cigarette smoking was not significantly associated with DPN (0.98 [0.75–1.29], *p* = 0.89).
**Cheng (2022) [27]**	Cohort Study	China	Overall: 1091; With DPN: 793; Without DPN: 298	Overall: 413:678; With DPN: 282:511; Without DPN: 131:167	With DPN: 60.40 ± 11.53; Without DPN: 54.79 ± 12.32	T2DM ± DPN	Smoking: Yes, No	Prevalence of smoking was significantly greater in those with DPN vs. those without (33.5% vs. 26.2%, *p* = 0.020), but was insignificant in multivariate analysis.
**Cho (2024) [28]**	Cohort Study	Korea	Overall: 2316; Non-smoker: 549; Ex-smoker: 924; Current Smoker: 843	0:2316	Non-smoker: 53.5 ± 11.3; Ex-smoker: 53.9 ± 9.7; Current Smoker: 49.4 ± 9.3	T2DM ± Ne	Smoking: Non, Ex, Current	Current smoking was significantly associated with increased odds of DN (1.38 [1.05–1.81], *p* = 0.021).
**Christensen (2020) [29]**	Cohort Study	Denmark	Overall: 5249; With DPoN: 938; With DPoN + Pain: 386	Overall: 2205:3144	^ 65 (57, 72)	T2DM ± DPoN ± Pain	Smoking: Never, Former, Current, Discontinued, Continued	Current and former smoking were significantly associated with DPoN (aPR = 1.50 [1.24–1.81]; aPR = 1.27 [1.06–1.52]). Current smoking was also significantly associated with NP in DoPN (aPR = 1.29 [1.03–1.62]).
**Kanbayashi (2022) [30]**	Cohort Study	Japan	Overall: 38; Data Points: 76	76:0	^^ CTCAE 0: 59 (45–73); 1: 58 (34–76); 2: 61 (40–66); 3: 66 (62–70)	Breast Cancer ± CIPN (Nab-paclitaxel)	Smoking History: Yes, No	Smoking history was significantly associated with CIPN on both CTCAE and PNQ (sensory only) during univariate (4.64 [1.60–13.5], *p* = 0.0048; 3.80 [1.40–10.30], *p* = 0.0087) and multivariate (4.79 [1.65–13.92], *p* = 0.004; 3.65 [1.38–9.69], *p* = 0.0093) analyses.
**Khan (2023) [31]**	Cohort Study	US	TUD: 8009; TAUD: 1672; PSUD: 642; TUD Co: 8009; TAUD Co: 1672; PSUD Co: 642	TUD: 4660:3349; TAUD: 582:1090; PSUD: 233:409; TUD Co: 4665:3344; TAUD Co: 584:1088; PSUD Co: 234:408	TUD: 61.6 ± 12.1; TAUD: 61.52 ± 10.3; PSUD: 57.84 ± 8.3; TUD Co: 61.6 ± 12.1; TAUD Co: 61.42 ± 10; PSUD Co: 57.88 ± 8.1	T2DM + Hypertension ± Neuropathy	TUD: Yes, No; TAUD: Yes, No; PSUD: Yes, No	DN was significantly higher in those with TUD vs. without (19.7% vs. 14.2%, 1.48 [1.35–1.61], *p* < 0.05).
**Kindl (2021) [32]**	Cohort Study	Germany	With MSK: 255; With CRPS: 223	With MSK: 160:95; With CRPS: 173:50	With MSK: 54.6 (20–80); With CRPS: 50.9 (18–77)	CRPS or MSK, due to trauma	Smoking: Yes, No	Smoking was significantly associated with higher current pain scores (NSRC: −0.252, SE: 0.118, SRC: −0.09, both *p* < 0.05)
**Lehtinen (1993) [33]**	Cohort Study	Finland	With ND: 12; Without ND: 101	With ND: 9:3; Without ND: 46:55	With ND: 57.2 ± 4.7; Without ND: 55.4 ± 10.4	DM ± ND	Smoking history	Smoking history was not significantly different between ND groups (25% vs. 48%, *p* > 0.05).
**Park (2023) [34]**	Cohort Study	Korea	Overall: 26,673; Never Smoked: 13,426 (Group 1: 8260; Group 2: 4106; Group 3: 1060); Quitting: 3426 (Group 4: 2848; Group 5: 578); Current Smokers: 9821 (Group 6: 2876; Group 7: 6945)	0:26,673	Overall: 59.6 ± 9; Group 1: 61.8 ± 9.4; Group 2: 61.3 ± 9.3; Group 3: 58.2 ± 8.9; Group 4: 58.7 ± 8.7; Group 5: 57.4 ± 8.3; Group 6: 57.8 ± 8.2; Group 7: 57.5 ± 8.1	T1DM or T2DM ± Ne	Smoking status: Group 1: never–never; Group 2: never–quitting; Group 3: never–current; Group 4: quitting–quitting; Group 5: quitting–current; Group 6: current–quitting; Group 7: current–current	Current–quitting smoking, current–current smoking, and heavy smoking (>20 pack-yrs) were significantly associated with DN (1.360 [1.076–1.719]; 1.237 [1.025–1.492]; 1.246 [1.048–1.481]).
**Sreeram (2023) [35]**	Cohort Study	US	Overall: 1034; With CIPN: 704; Without CIPN: 330	Overall: 797:237; With CIPN: 570:134; Without CIPN: 227:103	Overall: 57.1 ± 10.9 (27–79); With CIPN: 55.8 ± 10.8 (27–79); Without CIPN: 59.9 ± 10.4 (27–79)	Cancer survivors ± CIPN	Smoking (>100 cig/lifetime): Yes, No	Smoking was significantly different between CIPN groups (*p* = 0.004); however, it was not significantly associated with CIPN in multivariate analysis (1.20 [0.87–1.65], *p* = 0.27).
**Today Study Group (2022) [36]**	Cohort Study	US	Overall: 674; Normal MNSI: 528; Abnormal MNSI: 146; Normal Monofilament: 643; Abnormal Monofilament: 31	Normal MNSI: 171:357; Abnormal MNSI: 65:81; Normal Monofilament: 219:424; Abnormal Monofilament: 17:14	Normal MNSI: 13.9 ± 2; Abnormal MNSI: 14.3 ± 1.9; Normal Monofilament: 14 ± 2; Abnormal Monofilament: 14.2 ± 2.1	T2DM ± DPN	Smoking Status: Yes (within past mo), No (never or not within past mo)	Smoking was not significantly associated with DPN via abnormal MNSI (HR: 0.99 [0.83–1.65], *p* = 0.97) or monofilament (HR: 1.02 [0.33–3.17], *p* = 0.97).
**Voulgari (2011) [37]**	Cohort Study	Greece	193	97:96	56.4 ± 7.8	T2DM ± PN	Smoking: Cessation vs. Continuing	Smoking cessation was significantly associated with a reduction in PN at 1 yr (*p* < 0.05).
**Benbow (1997) [38]**	Case–Control Study	UK	With Pain: 49 (Current Smokers: 13; Ex-smokers: 15; Non-smokers: 21); Without Pain: 23	With Pain: 14:35 (Current Smokers: 4:9; Ex-smokers: 3:12; Non-smokers: 7:14); Without Pain: 9:14	With Pain: (Current Smokers: 54.2 ± 3.2; Ex-smokers: 57 ± 2.9; Non-smokers: 58 ± 2.9); Without Pain: 51 ± 1.9	DM ± PDN	Smoking: Current, Ex, Non; Pack-Yrs (20 cig/d for 1 yr = 1 pack-yr)	Current or past smoking was not significantly associated with the severity or duration of PDN (*p* > 0.05).
**Doneddu (2020) [39]**	Case–Control Study	Italy	Ca: 195; Co: 195	Ca: 109:86; Co: 109:86	NR	CIDP due to any etiology and their partners	Smoking Status: Yes (Current or Past), No	Smoking was not significantly associated with CIDP (1.43 [0.93–2.20], *p* = 0.1056).
**Fouchard (2023) [40]**	Case–Control Study	France	Overall: 323; Ca: 162; Co: 161	Overall: 192:131; Ca: 88:74; Co: 104:57	Ca: 56 ± 16; Co: 69 ± 13	Cutaneous paresthesia ± SFN via IENFD due to any etiology	Smoking: Yes, No	Smoking was significantly higher in those with SFN vs. without (26.5% vs.16.1%, *p* = 0.02).
**Franklin (1994) [41]**	Case–Control Study	US	Ca: 77; Co: 200	Ca: 29:48; Co: 118:82	Ca: 61.7; Co: 58.6	NIDDM ± DSN	Smoking: never, pack-years (<20, >20)	Smoking (pack-years: <20, >20) was not significantly associated with DSN (1.49 [0.69–3.22] *p* = 0.29, 0.74 [0.29–1.84]).
**Frost (2013) [42]**	Case–Control Study	Denmark	Ca: 324; Co: 832	Ca: 121:203; Co 317:515	Ca Smokers: 49 ± 9.7; Ca Non-smokers: 44 ± 11.5; Co Smokers: 50 ± 9; Co Non-smokers: 48 ± 9.9	Ca: Electroneurographically confirmed UN; Co: Without UN	Smoking: ever, never, pack-years	Pack-years (>10-<24 and >24) were significantly associated with UN (2.58 [1.48–4.51], 1.65 [1.37–1.99]). Current smoking was more significantly associated with UN (4.09 [2.43–6.86]) vs. ex-smokers (1.36 [0.83–2.23]), with highest pack-years exhibiting stronger associations in current (5.00 [2.69–9.32]) vs. ex-smokers (2.61 [1.16–5.88]). Smoking (>3 pack-years) was significantly associated with severe ulnar nerve damage, including localized demyelination (2.11 [0.95–4.72]) and axonal degeneration (1.65 [0.66–4.14]).
**Mitchell (1990) [43]**	Case–Control Study	US	IDDM: Ca: 54, Co: 56; NIDDM: Ca: 39, Co: 65	IDDM: Ca: 31:23, Co: 35:21; NIDDM: Ca: 25:14, Co: 44:21	IDDM: Ca: 36.1, Co: 32.7; NIDDM: 59.4: 39, Co: 57.7	DM ± Ne	Smoking: ever, median pack-yrs	Smoking (ever) was significantly higher in those with IDDM Ne vs. those without (64.8% vs. 42.8%, *p* < 0.05). Smoking (>30 median pack-yrs) was significantly associated with Ne in IDDM (40 vs. 0, *p* < 0.001; Mantel–Haenszel OR: 5.18 [1.91–14.1], *p* = 0.001; β = 1.2015, SE = 0.5397, 3.32 [1.15–9.58], *p* = 0.026).
**Mondelli (2020) [44]**	Case–Control Study	Italy	Ca: 220; Co: 460	Ca: 84:136; Co: 242:218	Ca: 51.7 ± 11.8; Co: 47.8 ± 12.4	Ca: UNE; Co: Upper-limb complaints	Tobacco: Pack-Yrs	Smoking (>25 pack-yrs) was significantly associated with both UNE and idiopathic UNE in univariate (3.3 [2–5.4]; 2.6 [1.3–5.2]) and multivariable (2.3 [1.3–4.1]; 2.2 [1–5.2]) analyses.
**Pessione (1995) [45]**	Case–Control Study	France	Ca: 32; Co: 58	Ca: 6:26; Co: 22:36	Ca: 49 ± 10.1; Co: 46.8 ± 9.6	Alcoholism ± PN	Smoking (current, former, never)	Tobacco consumption did not significantly differ between PN groups (*p* = 0.19).
**Richardson (2009) [46]**	Case–Control Study	US	Ca: 50; Co: 50	Ca: 18:32; Co: 34:16	Ca: 48.4 ± 12.8; Co: 39.2 ± 12	Ca: With UNE; Co: Without UNE	Smoking: ever, remote (quit >10 yrs), pack-years	Ever smoking and pack-yrs were significantly greater in those with UNE vs. without (25 vs. 15, *p* = 0.041; 0.29 ± 0.41 vs. 0.13 ± 0.27, *p* = 0.025). Pack-years were significantly associated with UNE (1.035 [1.001–1.070], *p* = 0.049). Electrophysiology including CMAP, CV, and conduction block were significantly associated with pack-yrs (*p* < 0.05).
**Abdissa (2020) [47]**	Cross-Sectional Study	Ethiopia	Overall: 366; With DPN: 196; Without DPN: 170	163:203	50.1 ± 14.28	T2DM ± DPN	Smoking: Current, Former, Never	Current and former smoking were both significantly associated with higher odds of DPN (7.96 [3.23–19.64], *p* < 0.001; 2.65 [1.22–5.77], *p* = 0.013).
**Alghamdi (2022) [48]**	Cross-Sectional Study	Saudi Arabia	Overall: 306; With Ne: 102; Without Ne: 102; Co: 102	Overall: 147:159; With Ne: 49:53; Without Ne: 49:53; Co: 49:53	With Ne: 54.1 ± 21.8; Without Ne: 54.1 ± 21.8; Co: 53.5 ± 13.1	T1DM or T2DM ± DSyPN	Smoking: Yes (Ever), No (Never)	Smoking was not significantly different between DSyPN groups (13.7% vs. 16.7%, *p* > 0.05).
**Asai (2022) [49]**	Cross-Sectional Study	Japan	Overall: 817; With CP: 35; Without CP: 782	Overall: 431:386; With CP: 24:11; Without CP: 407:375	With CP: 63.91 [60.11–67.72]; Without CP: 63.75 [63.02–67.72]	Chronic neck/shoulder/upper-limb pain due to any etiology	Current smoker: Yes, No	Current smoking was not significantly different between CP groups (20% vs. 18%, *p* = 0.77).
**Aubert (2014) [50]**	Cross-Sectional Study	France	198	40:158	65 (43–85)	T2DM ± PN	Current Smoking	Current smoking was significantly associated with greater PN severity when NDS was considered a continuous variable (*p* = 0.01) but was insignificant in univariate and multivariate models (*p* > 0.05).
**Billault (1991) [51]**	Cross-Sectional Study	France	157	45:55	41.6 ± 15.3	T1DM ± Ne	Tobacco Consumption: Pack-Yrs (20 cig/d for 1 yr = 1 pack-yr)	Tobacco consumption was not significantly different between Ne groups (11.98 ± 17.43 vs. 7.19 ± 12.93, *p* > 0.05).
**Callaghan (2020) [52]**	Cross-Sectional Study	US	BMI < 35 kg–Ne: 45; BMI > 35 kg–Ne: 110; BMI > 35 kg + Ne: 28	BMI < 35 kg–Ne: 37:8; BMI > 35 kg–Ne: 87:23; BMI > 35 kg + Ne: 18:10	BMI < 35 kg–Ne: 43.8 ± 12.1; BMI > 35 kg–Ne: 43.5 ± 11.2; BMI > 35 kg + Ne: 51.4 ± 9.6	Ca: BMI > 35 kg ± Ne; Co: BMI < 25 kg BMI < 35 kg–Ne; BMI > 35 kg–Ne; BMI > 35 kg + Ne	Tobacco: Current, Never, Former	Smoking status was not significantly different between Ne groups (BMI < 35 kg–Ne: 82.2% vs. BMI > 35 kg–Ne: 70.9% vs. BMI > 35 kg + Ne: 64.3%, *p* = 0.50).
**Çelik (2017) [53]**	Cross-Sectional Study	Turkey	444	190:254	F: 42.9 ± 11.2; M: 43.4 ± 14	NP due to any etiology	Smoking Duration (packs/yr)	Smoking duration was significantly higher in NP vs. without (31.8 ± 18.3 vs. 22.4 ± 15.5, *p* < 0.05), and addiction severity (on Fagerström scale) was significantly associated with pain existence (1.29 [1.14–1.46]).
**Chen (2024) [54]**	Cross-Sectional Study	China	Overall: 13,315; With DPN: 5847; Without DPN: 7468	With DPN: 3330:2517; Without DPN: 3774:3694	With DPN: 66.3 ± 9.8; Without DPN: 61 ± 11.4	T2DM ± DPN	Smoking: Yes (Ever), No (Never)	Smoking (ever vs. never) was not significantly associated with DPN (0.95 [0.91–1.01], *p* = 0.488).
**Chukwubuzo (2022) [55]**	Cross-Sectional Study	Nigeria	422	289:133	57.6 ± 10.1	T1DM or T2DM ± PN	Cigarette smoker: Yes, No	Smoking was not significantly associated with PDPN (0.81 [0.28–2.33], *p* = 0.7).
**Correa (2023) [56]**	Cross-Sectional Study	Brazil	Overall: 444; LLBP: 313; PNBP: 33; WP: 98	Overall: 289:155; LLBP: 188:125; PNBP: 26:7; WP: 75:23	Overall: 39.72 ± 14.68; LLBP: 37.02 ± 13.39; PNBP: 8.45 ± 14.30; WP: 48.78 ± 15.59	Chronic BP due to any etiology	Smoking: Yes, No	Smoking was not significantly associated with pain-related interference in BP (LLBP: 5.8% vs. PNBP: 9.1% vs. WP: 10.2%, *p* > 0.05).
**Dorsey (2009) [57]**	Cross-Sectional Study	US	948	LED+: 129:236; LED−: 302:281	!! LED+: 63.8 (0.92); LED−: 59.0 (0.54)	DM ± PN	Smoking: Never, Former, Current	Smoking status was not significant between LED groups (Never: 40.9%, Former: 33.8%, Current: 25.3% vs. 47.7%, 32.8%, 19.5%, *p* > 0.05).
**Faden (1981) [58]**	Cross-Sectional	USA	With COPD: 23; Without COPD: 8; Healthy Co: 12	NR	With COPD: 56.3 ± 6.7; Without COPD: 50.3 ± 8.7; Healthy Co: 30.4 ± 3.9	COPD ± PoN	Smoking: pack-yrs	Smoking was significantly greater in those with COPD vs. without (53.2 ± 8.76 vs. 11.1 ± 5.36, *p* < 0.0001), and was significantly associated with sural, ulnar, and radial sensory nerve dysfunction in COPD (rs = 0.68, *p* < 0.01; rs = 0.48, *p* < 0.01; rs = 0.38, *p* < 0.05).
**Gode (2022) [59]**	Cross-Sectional Study	Ethiopia	Overall: 216; With PN: 108; Without PN: 108	Overall: 111:105; With PN: 54:54; Without PN: 57:51	Overall: 57.9 ± 12.6; With PN: 62.2 ± 10.9; Without PN: 53.5 ± 12.7	T2DM ± PN	Smoking: Yes, No	Cigarette smoking did not significantly differ between PN groups (28.6% vs. 71.4%, *p* = 0.249); very small sample sizes.
**Gunduz (2022) [60]**	Cross-Sectional Study	Turkey	Overall: 109; LANSS > 12: 8	Overall: 41:68; LANSS > 12: 4:4	Overall: 54 ± 13.97; LANSS > 12: 47.5 ± 10.6	Thoracotomy ± NP	Smoking History: Pack/Yr	Smoking history was significant between LANSS > 12 vs. overall (21.8 ± 19.7 vs. 33.1 ± 14.1, *p* = 0.05).
**Gylfadottir (2020) [61]**	Cross-Sectional Study	Denmark	5514	2355:3159	64.1 ± 10.9	T2DM ± DPoN	Smoking: Active, Daily, Occasionally, Previous, Never	Smoking (ever vs. never) was significantly associated with DPoN (1.36 [1.14–1.63], *p* < 0.05) and painful DPoN (1.52 [1.20–1.93], *p* < 0.05).
**Hicks (2022) [62]**	Cross-Sectional Study	US	Overall: 6902; With PN: 1181; Without PN: 5721	Overall: 3589:3313; With PN: 443:738; Without PN: 3101:2620	%! 40–49: 36 (0.9); 50–59: 27.8 (0.8); 60–69: 18.2 (0.6); 70–79: 12.8 (0.4); ≥80: 5.2 (0.3)	DM ± PN	Tobacco: Never, Former, Current	Tobacco use reported between PN groups: Never: 43.4%, Former: 38.6%, Current: 18% vs. 46.8%, 32.5%, 20.7% (statistics NR).
**Jaiswal (2017) [63]**	Cross-Sectional Study	US	1992	1037:955	(14–27)	T1DM or T2DM ± DPN	Smoking: Non, Former, Current	Smoking was significantly higher in those with DPN vs. without in both T1DM (54% vs. 31%, *p* = 0.001) and T2DM (72% vs. 58%, *p* = 0.01).
**Jeyam (2020) [64]**	Cross-Sectional Study	Scotland	Overall: 5558; With DPN 715; Without DPN 4842	Overall: 2449:3109; With DPN: 320:395; Without DPN 2129:2713	^ Overall: 44.7 (33, 55.2); With DPN: 50.6 (41, 59.3); Without DPN: 43.7 (32, 54.4)	T1DM ± DPN	Smoking: Ever, Never	Smoking (ever) was significantly associated with DPN (1.67 [1.37–2.03], *p* < 0.05).
**Kowalski (2022) [65]**	Cross-Sectional Study	US	Overall: 43; With NP: 28; Without NP: 15	With NP: 4:24; Without NP: 4:11	Overall: 38.65 ± 12.12; With NP: 40.12 ± 12.77; Without NP: 35.91 ± 10.67	SCI ± NP	Smoking: Ever (1+/d for 1 yr or 20+ packs in a lifetime), Never	NP severity was significantly greater in those with a history of smoking vs. without (6.77 ± 0.65 vs. 4.65 ± 0.69, *p* = 0.04).
**Li (2023) [66]**	Cross-Sectional Study	China	Overall: 25,710; With PDPN: 14,699; Without PDPN: 11,011	Overall: 10,785:14,925; With PDPN: 6240:8459; Without PDPN: 4545:6466	^ Overall: 63 (55, 71); With PDPN: 65 (56, 73); Without PDPN: 61 (53, 69)	T2DM ± PDPN	Smoking: Yes, No	PDPN was significantly higher in smokers vs. non-smokers (54.8% vs. 45.2%, *p* = 0.001); however, smoking was reported less among those with PDPN compared to those without (16.2% vs. 17.9%, *p* = 0.001).
**Al-Mahroos (2007) [67]**	Cross-Sectional Study	Bahrain	1477	842:635	57.3 ± 6.32	T2DM ± DPN	Smoking: Yes, No	DPN was significantly higher in smokers vs. non-smokers (57% vs. 16%, *p* = 0.05). Smoking status was significantly associated with DPN in univariate and multivariate analyses (2.18 [1.16–1.77], *p* = 0.002; 1.24 [1.01–1.31], *p* < 0.02).
**Mick (2021) [68]**	Cross-Sectional Study	France, Italy, Spain, UK	1030	651:379	60.2 ± 15.32; ^ 61 (49–72)	Localized NP due to any etiology	Tobacco: Ex, Never, Current (≤10 yrs, >10 yrs, <10 cig/d, ≥10 cig/d	Never smoking was more frequently reported in NP (59.2%) compared to ex- and current smokers (20.55% vs. 20.25%); additional statistics for NR.
**Mizokami-Stout (2020) [69]**	Cross-Sectional Study	US	Overall: 5936; With DPN: 630; Without DPN: 5306	Overall: 3265:2671; With DPN: 378:252; Without DPN: 2865:2441	Overall: 39 ± 18; With DPN 51 ± 17; Without DPN: 37 ± 17	T1DM ± DPN	Smoking: Yes, No	Smoking was significantly different between DPN groups (8% vs. 4%, *p* = 0.008), and was significantly associated with DPN (1.83 [1.18–2.82]).
**Molla (2020) [70]**	Cross-Sectional Study	Iran	Overall: 99,651; With T1DM: 10,390; With T2DM: 82,308	68,743:30,424	57.4 ± 12.8	T1DM or T2DM ± Ne	Smoking: Yes (Current), No (Past/Never)	Smoking was significantly associated with Ne in both T1DM and T2DM (2.40 [1.95–2.96], *p* < 0.001; 1.70 [1.69–1.93], *p* < 0.001).
**Moore (2019) [71]**	Cross-Sectional Study	US	Normal BMI (<25): 52; Overweight BMI (25–29.9): 52; Obese BMI (>30): 39	Normal BMI (<25): 19:33; Overweight BMI (25–29.9): 19:33; Obese BMI (>30): 21:18	^^ Normal BMI (<25): 66 (41–81); Overweight BMI (25–29.9): 69.5 (42–85); Obese BMI (>30): 61 (25–81)	Newly Diagnosed Multiple Myeloma ± CIPN (Bortezomib)	Smoking: Never, Former, Current	Former vs never, and current vs. never smoking were not significantly associated with CIPN (0.613 [0.285–1.317], *p* = 0.45; 0.750 [0.179–3.134], *p* = 0.45).
**Nagakura (2023) [72]**	Ecological Cross-Sectional Study	Japan	Pregabalin Reimbursement Claims per 1000 population; up to 126 million	NR	(40–74)	NP due to any etiology treated with pregabalin	Tobacco (>100 cig or >6 mo): Yes, No	Smoking was significantly associated with an increase prevalence of NP (β = 206.4 [45–367.8], *p* = 0.0134).
**Nielsen (2022) [73]**	Cross-Sectional Study	Denmark	2839	High CIPN Score: 274:146; Low CIPN Score: 1193:870	^^ High CIPN Score: 69; Low CIPN Score: 67; (18–99)	Cancer diagnosis at any stage of treatment ± CIPN	Tobacco: yes/no	Smoking was significantly associated with higher CIPN20 scores vs. lower scores (21% vs. 15%, *p* = 0.04).
**Ponirakis (2022) [74]**	Cross-Sectional Study	Qatar, Kuwait and KSA	Overall: 3021; Qatar: 1093; Kuwait: 1168; KSA: 760	Overall: 1408:1613; Qatar: 428:665; Kuwait: 552:616; KSA: 428:332	Overall: 57.9 ± 11.7; Qatar: 52.4 ± 11.3; Kuwait: 63.3 ± 9.9; KSA: 57.1 ± 11.2	T2DM + DPN or NP	Smoking: Yes (1+/d over 1 yr), No	Smoking was significantly associated with PN (1.5 [1.2–1.9], *p* < 0.01) overall, and painful PN (1.8 [1.1–3.0], *p* = 0.01) in Qatar only.
**Rash (2022) [75]**	Cross-Sectional Study	US	Overall: 933; Current Smokers: 166; Former Smokers: 252; Never Smokers: 515	Overall: 569:364; Current Smokers: 61:105; Former Smokers: 146:106; Never Smokers: 361:154	Overall: 38 ± 16; Current Smokers: 31 ± 12; Former Smokers: 46 ± 15; Never Smokers: 37 ± 16	T1DM ± Ne	Smoking: Never, Former, Current (Daily, <Daily)	Ne was significantly higher in daily smokers compared to < daily smokers (18% vs. 11%, *p* = 0.09)
**Revesz (2022) [76]**	Cross-Sectional Study	The Netherlands	Overall: 1516; With PN: 980; Without PN: 536	Overall: 634:882; With PN: 445:535; Without PN: 189:347	Overall: 69.1 ± 9.4; With PN: 70.1 ± 9.4; Without PN: 67.2 ± 9.2	Colorectal cancer survivors ± PN	Smoking: Never, Former, Current	Smoking was significantly associated with higher motor, autonomic, and total PN
**Sahito (2022) [77]**	Cross-Sectional Study	Pakistan	Overall: 1057; With PN: 607; Without PN: 450	Overall: 414:643; With PN: 230:377; Without PN: 184:266	30–40: 119; 41–50: 316; 51–60: 324; 61–70: 165; >70 yrs: 133	T2DM ± PN	History of smoking: Yes, No	Smoking was significantly higher in those with DPN vs. without (41.8% vs. 37.2%, statistics NR).
**Srivastava (2022) [78]**	Cross-Sectional Study	India	98	79:19	51.63 ± 10.68	Cancer survivors ± CIPN	History of smoking: Yes (current, former), No	Smoking history was not significantly associated with sensory, motor, or autonomic CIPN severity (*p* > 0.05).
**Tamer (2005) [79]**	Cross-Sectional Study	Turkey	191	109:82	58.7 ± 10	T2DM ± DSPoN	Smoking: Yes, No	Smoking was significantly associated with electromyography supported DSPoN (2.330 [1.222–4.445]).
**Tesfaye (1996) [80]**	Cross-Sectional Study	Europe	3250	1582:1668	32.7 ± 10.2 (15–61)	IDDM ± DPN	Smoking (current, former, never)	Current smoking was significantly associated with DPN (RR: 2.4 [1.1–5], *p* = 0.02) in multivariate analysis.
**Trendowski (2021) [81]**	Cross-Sectional Study	US	With CIPN: 550; Without CIPN: 495	With CIPN: 440:110; Without CIPN: 355:140	^^ With CIPN: 56 (23–79); Without CIPN: 58 (21–79)	African American cancer survivors ± CIPN	Tobacco: Never, Ever (≥100 cig/lifetime), Current; Smoking Frequency (cig/d): 1–9, 10–19, ≥20	Ever and current smoking were not significantly associated with CIPN (1.04 [0.80–1.35], *p* = 0.76; 1.28 [0.90–1.82], *p* = 0.18).
**Van der Velde (2019) [82]**	Cross-Sectional Study	The Netherlands	Overall: 2401; High Sural SNAPA: 793; Med Sural SNAPA: 796; Low Sural SNAPA 812	Overall: 1174:1227; High Sural SNAPA: 464:329; Med Sural SNAPA: 377:419; Low Sural SNAPA: 334:478	Overall: 59.3 ± 8.2; High Sural SNAPA: 56.4 ± 8.2; Med Sural SNAPA: 59.4 ± 7.9; Low Sural SNAPA: 62 ± 7.5	T2DM ± PN	Smoking: Current, Former, Never	Current smoking was associated with worse nerve function (β = −0.11 [−0.17–−0.04]) and higher VPT (β = 0.17 [0.06–0.28]). Former smoking was associated with lower peroneal NCV (β = −0.12 [−0.20–−0.05]). Smoking (ever) was associated with NP (2.13 [1.38–3.29]).
**Wang (2023) [83]**	Cross-Sectional Study	China	Overall: 14,908; With DPN: 10,084; Without DPN: 4824	Overall: 6322:8586; With DPN: 4365:5719; Without DPN: 1957:2867	Overall: 61.3 ± 13, ^ 62 (53, 70); With DPN: 62.6 ± 12.5, ^ 63 (55, 71); Without DPN: 58.5 ± 13.5, ^ 59 (50, 67)	T2DM ± DPN	Tobacco: Current	Smoking was significantly lower in those with DPN compared to without (14.9% vs. 20.7%, *p* < 0.001).
**Weinberger (2018) [84]**	Cross-Sectional Study	US	Overall: 103; With Pain: 70; Without Pain: 33	With Pain: 34:36; Without Pain: 17:16	With Pain: 50.5 ± 8.2; Without Pain: 47.7 ± 9.7	HIV/AIDS ± NP	Smoking habits: daily vs. <daily, cig/d (>10 vs. <10), Quit attempts (number and d of longest)	All smoking habits were not significantly associated with NP (*p* > 0.05).
**Yokoyama (2020) [85]**	Cross-Sectional Study	Japan	Overall: 9914; Without DPoN: 6180; With DPoN: 2745 (with DPoNS: 1689; with UDoPN: 989)	Overall: 3715:6139; Without DPoN: 2273:3904; With DPoN: 1041:1705 (with DPoNS: 664:1025, with UDPoN: 397:530)	^^ Overall: 66 (69–73); Without DPoN: 65 (57–71); With DPoN: 70 (63–77) (with DPoNS: 69 (63–76), with UDPoN: 67 (59–75))	T2DM ± DPoN	Tobacco: Current, Former, Never	Current and former smoking were significantly associated with DPoN (2.04 [1.42–2.91], *p* < 0.001; 1.64 [1.19–2.26], *p* = 0.002).
**Richards (2005) [86]**	Case Series	US	2	0:2	38 and 55	SCI + NP	Smoking abstinence prior to surgery	Subjective improvement of NP during smoking abstinence, and NP returned when smoking re-endorsed (additional statistics NR).

**aPR:** adjusted prevalence ratio; **BMI:** body mass index; **Ca:** cases; **CIDP:** chronic inflammatory demyelinating polyradiculoneuropathy defined via EFNS/PNS 2010 criteria; **Cig:** cigarette; **CIPN:** chemotherapy-induced peripheral neuropathy; **CIPN20:** European Organization for Research and Treatment of Cancer CIPN 20-item scale; **CMAP:** compound muscle action potential; **Co:** controls; **COPD:** chronic obstructive pulmonary disease; **CP:** chronic pain; **CRPS:** complex regional pain syndrome defined via Budapest criteria; **CTCAE:** National Cancer Institute Common Terminology Criteria for Adverse Events; **CV:** conduction velocity; **d:** day; **DM:** diabetes mellitus; **DN:** diabetic neuropathy; **DPN:** diabetic peripheral neuropathy; **DPoN:** diabetic polyneuropathy; **DPoNS:** diabetic polyneuropathy-related sensory symptoms/signs; **DSN:** distal symmetric neuropathy; **DSPoN:** distal sensory polyneuropathy; **DSyPN:** distal symmetrical peripheral neuropathy; **F:** female; **HR:** hazard ratio; **IDDM:** insulin-dependent diabetes mellitus; **IEFND:** intraepidermal nerve fiber density; **kg:** kilogram; **KSA:** Kingdom of Saudi Arabia; **LANSS:** Leeds assessment of neuropathic symptoms and signs; **LED:** lower-extremity disease (includes both peripheral neuropathy and peripheral arterial disease); **LLBP:** localized lower-back pain; **M:** male; **MNSI:** Michigan neuropathy screening instrument; **mo:** month; **MSK:** musculoskeletal pain; **NCV:** nerve conduction study; **NDS:** neuropathy disability score; **ND:** neurophysiologically deteriorated; **Ne:** neuropathy; **NIDDM:** non-insulin-dependent diabetes mellitus; **NP:** neuropathic pain; **NR:** not reported; **NSRC:** not standardized regression coefficient; **PDN:** painful diabetic neuropathy; **PDPN:** painful diabetic peripheral neuropathy; **PN:** peripheral neuropathy; **PNBP:** peripheral neuropathic back pain; **PNQ:** patient neurotoxicity questionnaire; **PoN:** polyneuropathy; **PSUD:** polysubstance use disorder group (tobacco, alcohol, + 1 other); **PY:** person-years; **RR:** relative risk; **rs:** Spearman rank correlation test; **SCI:** spinal cord injury; **SE:** standard error; **SFN:** small-fiber neuropathy; **SNAPA:** sensory nerve action potential amplitude; **SRC:** standardized regression coefficient; **T1DM:** type 1 diabetes mellitus; **T2DM:** type 2 diabetes mellitus; **TAUD:** tobacco and alcohol use disorder group; **TUD:** tobacco use disorder group; **UDPoN:** unknown-status diabetic polyneuropathy; **UN:** ulnar neuropathy; **UNE:** ulnar neuropathy at the elbow; **VPT:** vibration perception threshold; **WP:** widespread pain; **yr (s):** year(s); **β:** beta coefficient; **^:** median (interquartile range); **^^:** median (range); **!:** SE; **!!:** mean (standard error); *All data reported as mean ± SD, mean (range), or mean [95% CI]; outcome data reported as* cases vs. controls, *or as “with* Ne” vs. “without *Ne”, with OR [95% CI], p-value (adjusted p-value when available), unless otherwise specified.*

### 3.2. Included Studies

All studies included in this systematic review were observational. Data from 49,856 participants from 13 cohort studies were included, with sample sizes ranging from 38 to 26,673 individuals per study [25,26,27,28,29,30,31,32,33,34,35,36,37]. The age of participants across all cohort studies varied between 10 and 79 years, with 20.71% being female. Geographic representation was limited to high-income countries, as defined by the World Bank, with studies published between 1993 and 2024. Most cohort studies focused on populations with peripheral neuropathy (PN) (n = 6) [26,27,30,35,36,37], or broadly defined neuropathy (n = 5) [25,28,31,33,34], with fewer examining pain (n = 1) [25] and complex regional pain syndrome (CRPS) (n = 1) [32]. Neuropathy etiologies were predominantly diabetes mellitus (n = 10) [25,26,27,28,29,31,33,34,36,37], as well as cancer (n = 2) [30,35] and trauma (n = 1) [32]. Cohort studies all reported neuropathy status in relation to smoking exposure, categorized as any consumption (n = 10) [25,26,27,28,29,30,32,33,34,36], smoking dependence (n = 1) [31], lifetime smoking (n = 1) [35], or smoking cessation (n = 1) [37] (Table 1). However, only 12 [25,26,28,29,30,31,32,33,34,35,36,37] studies provided sufficiently granular data to calculate odds ratios and relative risks for neuropathy incidence among exposed versus non-exposed groups (Table 2).

A total of 1202 cases and 2100 controls from nine case–control studies were included, with sample sizes ranging from 72 to 1156 individuals per study [38,39,40,41,42,43,44,45,46]. The mean age of participants varied between 32.7 and 69 years (±13), with 47.21% being female. Studies were published from 1990 to 2023 and were all conducted in high-income countries. Ulnar neuropathy of the elbow (UNE) (n = 3) [42,44,46] was the most commonly studied condition, followed by chronic inflammatory demyelinating polyradiculoneuropathy (CIDP), small-fiber neuropathy (SFN), distal symmetric neuropathy (DSN), PN, painful diabetic neuropathy (PDN), and general neuropathy (n = 1 each) [38,39,40,41,43,45], attributed to any etiology (n = 5) [39,40,42,44,45], diabetes mellitus (n = 3) [38,41,43], or alcohol dependence (n = 1) [45]. Smoking exposure was categorized as any consumption (n = 9) [38,39,40,41,42,43,44,45,46] (Table 1)**.** Eight studies [39,40,41,42,43,44,45,46] reported sufficient granular data to calculate odds ratios for the association between smoking exposure and neuropathy (Table 2).

Overall, 39 cross-sectional studies, comprising 213,195 participants, were included, with sample sizes ranging from 43 to 99,651 individuals per study [47,48,49,50,51,52,53,54,55,56,57,58,59,60,61,62,63,64,65,66,67,68,69,70,71,72,73,74,75,76,77,78,79,80,81,82,83,84,85]. Participant ages ranged from 14 to over 80 years old, with 57.27% females across all studies. One ecological cross-sectional study specifically examined insurance reimbursement claims per 1000 of the population, up to 126 million individuals, and was therefore not included in these averages [72]. All cross-sectional studies were published between 1981 and 2024 and were conducted in both high- (34/39) [48,49,50,51,52,53,54,56,57,58,60,61,62,63,64,65,66,67,68,69,70,71,72,73,74,75,76,79,80,81,82,83,84,85] and low-income (5/39) [47,55,59,77,78] countries. PN accounted for over half of the diagnoses across all studies (n = 22) [47,50,54,55,57,59,62,63,64,66,67,69,71,73,74,76,77,78,80,81,82,83], followed by painful neuropathy (n = 6) [53,60,65,68,72,84], polyneuropathy (PoN) (n = 5) [48,58,61,79,85], general neuropathy (n = 4) [51,52,70,75], and pain (n = 2) [49,56] to a lesser extent. Diabetes mellitus (n = 24) [47,48,50,51,54,55,57,59,61,62,63,64,66,67,69,70,74,75,77,79,80,82,83,85] was the most frequently reported etiology, followed by any cause (n = 5) [49,53,56,68,72], cancer (n = 5) [71,73,76,78,81], BMI, chronic obstructive pulmonary disease (COPD), HIV, surgery, and trauma (n = 1 each) [52,58,60,65,84]. Smoking exposure was categorized as any consumption (n = 38) [47,48,49,50,51,52,53,54,55,56,57,58,59,60,61,62,63,64,65,66,67,68,69,70,71,72,73,74,75,76,77,78,79,80,81,82,83,85] and daily smoking quantity (n = 1) [84] (Table 1). Prevalence estimates of neuropathy were reported in relation to smoking exposure in 24 studies [47,48,49,50,52,54,56,57,59,61,62,63,64,65,70,73,75,77,79,80,82,83,84,85], with odds ratios used to quantify the strength of these associations (Table 2). Lastly, one case series, comprising two male individuals aged 38 and 55 years, examined smoking cessation for neuropathic pain, following surgery for spinal cord injury [86] (Table 1).

### 3.3. Risk of Bias

**Figure 2:**
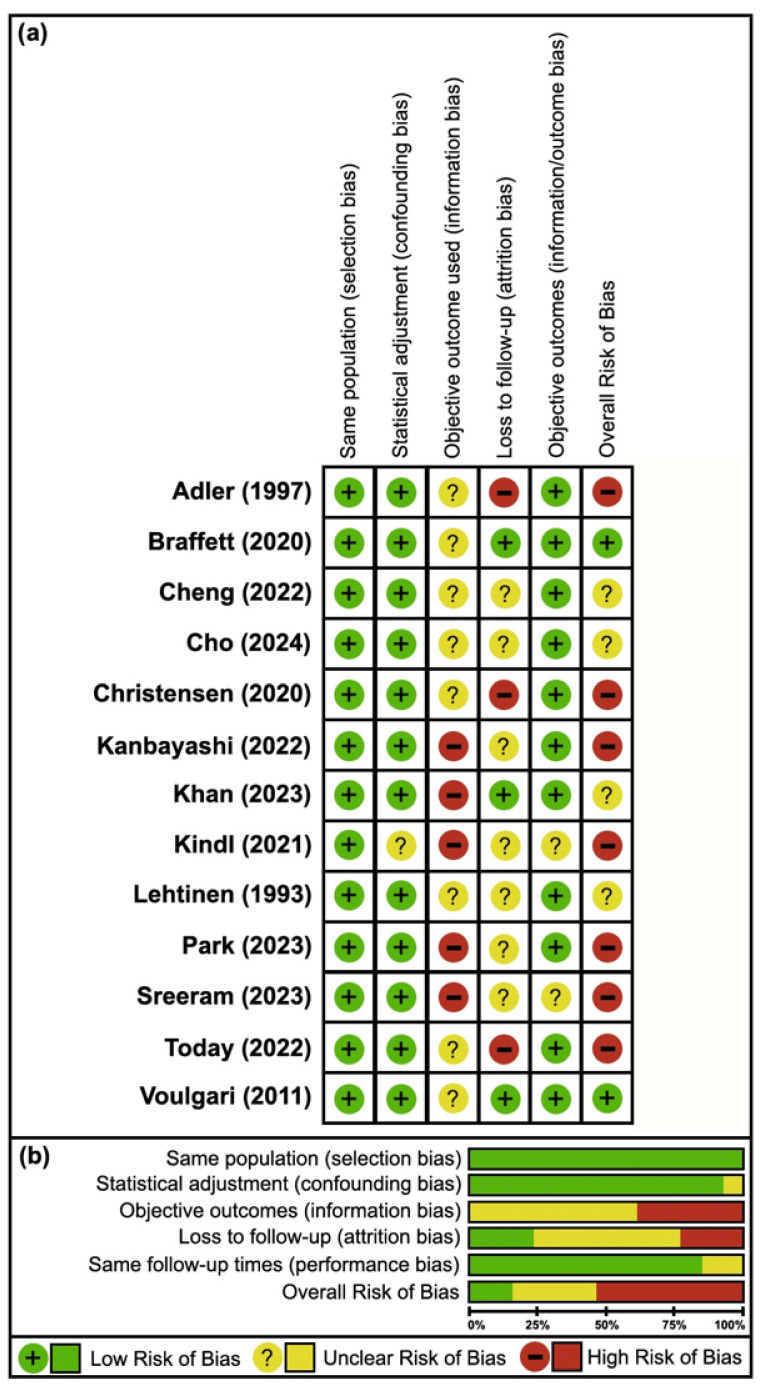
Risk-of-bias assessment for cohort studies. (**a**) Risk-of-bias summary by cohort study; (**b**) summary of risk-of-bias items by bias item. Included studies: [25,26,27,28,29,30,31,32,33,34,35,36,37].

The risk of bias among cohort studies was generally moderate, with low risk observed across 60% (39/65) of all assessed measures. Major information and attrition biases were present, as all studies failed to utilize objective outcomes (13/13, 100%) [25,26,27,28,29,30,31,32,33,34,35,36,37] and reported moderate to high loss to follow-up (10/13, 77%) [25,27,28,29,30,32,33,34,35,36], while selection, confounding, and outcome biases were relatively low overall (0/13, 0%; 1/13, 8% [32]; 2/13, 15% [32,35], respectively) (Figure 2).

**Table 2:** Summary of findings.

Incidence of Neuropathy/NP With/Without Smoking											
**Population(s):** DM ± PN; DM ± Ne; CRPS or MSK; T2DM ± DPoN ± Pain; CIPN; DM ± ND											
**Exposure:** Smoking											
**Comparison:** No smoking											
**Outcome:** Neuropathy incidence											
**Setting:** Denmark, Finland, Germany, Japan, Korea, the USA											
**Study Design:** Cohort studies											
**Stratification**	**No. of studies**	**Neuropathy-positive (%)**	**Neuropathy-negative (%)**	**Odds ratio** **(95% CI)**	**Relative risk** **(95% CI)**	**Risk of bias**	**Inc**	**Ind**	**Imp**	**Certainty of evidence (GRADE)**	**References**
Current/Former Smoking	9	1734/2957 (58.64%)	19,303/29,261 (65.97%)	0.73 (0.68–0.79)	0.89 (0.86–0.92)	Very serious	Low risk	Low risk	Low risk	Very Low ⨁**◯◯◯**	Braffett (2020) [26], Cho (2024) [28], Kindl (2021) [32], Christensen (2020) [29], Park (2023) [34], TODAY (2022) [36], Kanbayashi (2022) [30], Adler (1997) [25], Lehtinen (1993) [33]
Smoking Dependence	1	2129/3266 (65.19%)	8194/15,066 (54.39%)	1.57 (1.45–1.70)	1.20 (1.16–1.23)	Serious	NA	Low risk	Low risk	Very Low ⨁**◯◯◯**	Khan (2023) [31]
Lifetime Smoking	1	328/700 (46.86%)	185/328 (56.40%)	0.68 (0.52–0.89)	0.83 (0.73–0.94)	Very serious	NA	Low risk	Low risk	Very Low ⨁**◯◯◯**	Sreeram (2023) [35]
Smoking Cessation	1	22/35 (62.86%)	98/158 (62.03%)	1.04 (0.51–2.16)	1.01 (0.76–1.34)	Not serious	NA	Low risk	High risk	Very Low ⨁**◯◯◯**	Voulgari (2011) [37]
**Association Between Neuropathy/NP and Smoking**											
**Population:** CIDP; Paresthesia ± SFN; UNE; NIDDM ± DSN; Alcoholism ± PN; DM ± Ne; healthy controls											
**Exposure:** Smoking											
**Comparison:** No smoking											
**Outcome:** Neuropathy association											
**Setting:** Denmark, France, Italy, US											
**Study Design:** Case–control studies											
**Stratification**	**No. of studies**	**Neuropathy-positive (%)**	**Neuropathy-negative (%)**	**Odds ratio** **(95% CI)**	**Relative risk** **(95% CI)**	**Risk of bias**	**Inc**	**Ind**	**Imp**	**Certainty of evidence (GRADE)**	**References**
Current/Former Smoking	8	664/1153 (57.59%)	1007/2075 (48.53%)	1.44 (1.25–1.67)	---	Serious	Low risk	Low risk	Low risk	Very Low ⨁**◯◯◯**	Doneddu (2020) [39], Fouchard (2023) [40], Mondelli (2020) [44], Richardson (2009) [46], Frost (2013) [42], Franklin (1994) [41], Pessione (1995) [45], Mitchell (1990) [43]
**Prevalence of Neuropathy/NP with/without Smoking**											
**Population:** DM ± PN; CIPN; T2DM ± DPoN; SCI ± NP; Chronic BP; T2DM ± DSPoN; BMI > 35 kg ± Ne; Chronic neck/shoulder/upper-limb pain; DM ± DSyPN; IDDM ± DPN; HIV/AIDS ± NP											
**Exposure:** Smoking											
**Comparison:** No smoking											
**Outcome:** Neuropathy prevalence											
**Setting:** Brazil, China, Denmark, Ethiopia, Europe, France, Iran, Japan, the Netherlands, Pakistan, Saudi Arabia, Scotland, Turkey, the US											
**Study Design:** Cross-sectional studies											
**Stratification**	**No. of studies**	**Neuropathy-positive (%)**	**Neuropathy-negative (%)**	**Odds ratio** **(95% CI)**	**Relative risk** **(95% CI)**	**Risk of bias**	**Inc**	**Ind**	**Imp**	**Certainty of evidence (GRADE)**	**References**
Current/Former Smoking	23	9238/52,595 (17.56%)	18,298/114,710 (15.95%)	1.12 (1.09–1.15)	---	Not serious	High risk	Mod risk	Low risk	Very Low ⨁**◯◯◯**	Sahito (2022) [77], Abdissa (2020) [47], Rash (2022) [75], Nielsen (2022) [73], Gylfadottir (2020) [61], Kowalski (2022) [65], Jeyam (2020) [64], Dorsey (2009) [57], Jaiswal (2017) [63], Molla (2020) [70], Chen (2024) [54], Correa (2023) [56], Gode (2022) [59], Tamer (2005) [79], Wang (2023) [83], Yokoyama (2020) [85], Callaghan (2020) [52], Hicks (2022) [62], Asai (2022) [49], Aubert (2014) [50], van der Velde (2019) [82], Alghamdi (2022) [48], Tesfaye (1996) [80]
Lifetime Smoking	1	32/66 (48.49%)	15/33 (45.46%)	1.13 (0.50–2.64)	---	Very serious	NA	Low risk	High risk	Very Low ⨁**◯◯◯**	Weinberger (2018) [84]

**BP:** back pain; **CI:** confidence interval; **CIDP:** chronic inflammatory demyelinating polyradiculoneuropathy; **CIPN:** chemotherapy-induced peripheral neuropathy; **CRPS:** complex regional pain syndrome; **DM:** diabetes mellitus; **DPN:** diabetic peripheral neuropathy; **DPoN:** diabetic polyneuropathy; **DSN:** distal symmetric neuropathy; **DSPoN:** distal sensory polyneuropathy; **DSyPN:** distal symmetrical peripheral neuropathy; **IDDM:** insulin-dependent diabetes mellitus; **Imp:** imprecision; **Inc:** inconsistency; **Ind:** indirectness; **MSK:** musculoskeletal pain; **ND:** neurophysiologically deteriorated; **Ne:** neuropathy; **NIDDM:** non-insulin-dependent diabetes mellitus; **NP:** neuropathic pain; **PN:** peripheral neuropathy; **SCI:** spinal cord injury; **SFN:** small-fiber neuropathy; **T2DM:** type 2 diabetes mellitus; **UNE:** ulnar neuropathy at the elbow. ***GRADE Working Group grades of evidence: high certainty*** *(we are very confident that the true effect lies close to that of the estimate of the effect); **moderate certainty** (we are moderately confident in the effect estimate—the true effect is likely to be close to the estimate of the effect, but there is a possibility that it is substantially different); **low certainty** (our confidence in the effect estimate is limited—the true effect may be substantially different from the estimate of the effect); and **very low certainty** (we have very little confidence in the effect estimate—the true effect is likely to be substantially different from the estimate of effect).*

**Figure 3:**
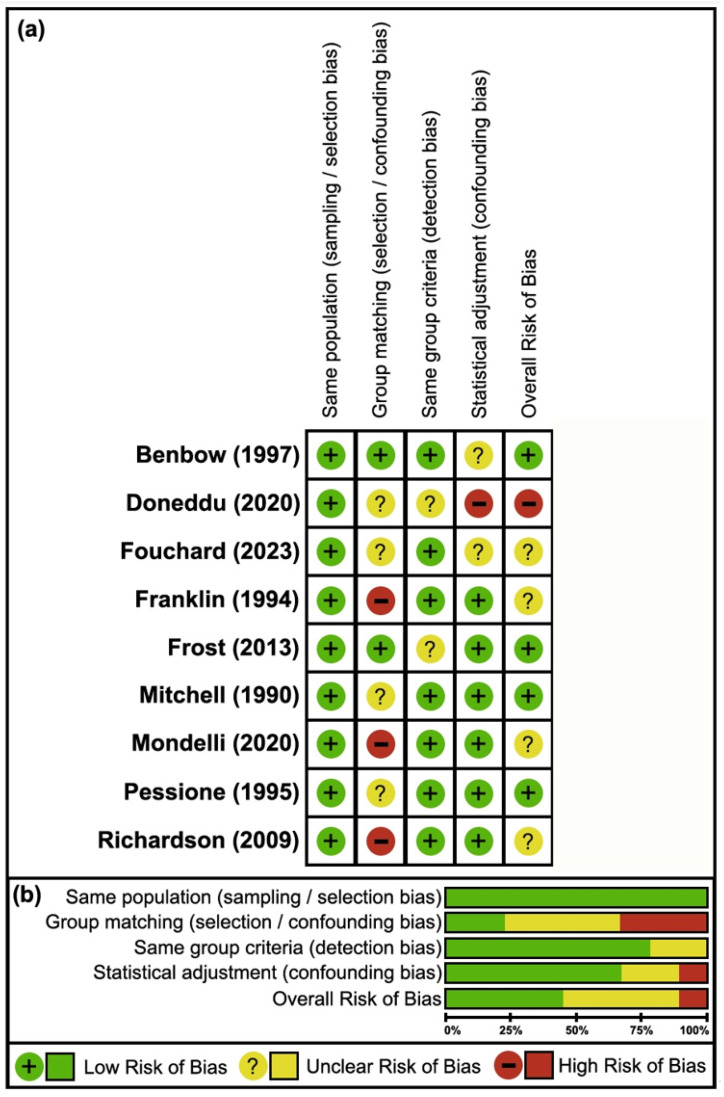
Risk-of-bias assessment for case–control studies. (**a**) Risk-of-bias summary by case–control study; (**b**) summary of risk-of-bias items by bias item. Included studies: [38,39,40,41,42,43,44,45,46].

For case–control studies, the overall risk of bias was also moderate, with 67% (24/36) of measures classified as low risk. Although sampling/selection bias was not detected in any of the studies (0/9, 0%), significant bias arose from inadequate matching of cases and controls in most studies (7/9, 78%) [39,40,41,43,44,45,46]. Detection and confounding biases were less frequent (2/9, 22% [39,42]; 3/9, 33% [38,39,40], respectively) as well (Figure 3).

**Figure 4:**
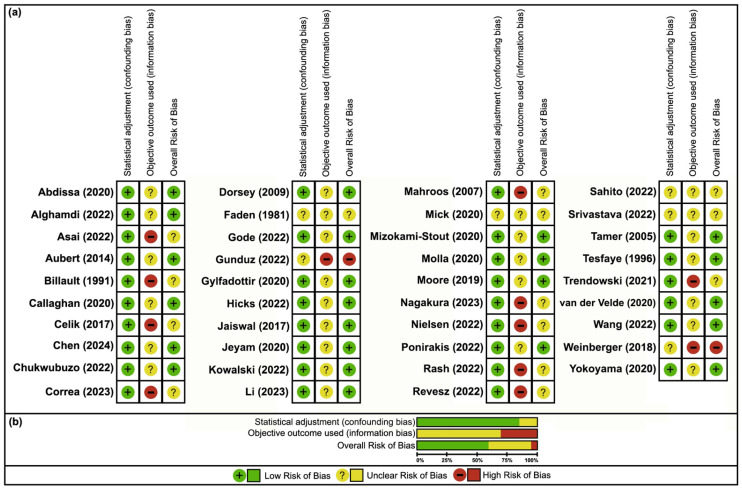
Risk-of-bias assessment for cross-sectional studies. (**a**) Risk-of-bias summary by cross-sectional study; (**b**) summary of risk-of-bias items by bias item. Included studies: [47,48,49,50,51,52,53,54,55,56,57,58,59,60,61,62,63,64,65,66,67,68,69,70,71,72,73,74,75,76,77,78,79,80,81,82,83,84,85].

Similarly, cross-sectional studies also exhibited an overall moderate risk of bias, with 42% (33/78) of all measures classified as low risk. While statistical adjustments were adequate in many of the studies (33/39, 85%) [47,48,49,50,51,52,53,54,55,56,57,59,61,62,63,64,65,66,67,69,70,71,72,73,74,75,76,79,80,81,82,83,85], profound information bias was evident, as all studies failed to use objective outcomes (39/39, 100%) [47,48,49,50,51,52,53,54,55,56,57,58,59,60,61,62,63,64,65,66,67,68,69,70,71,72,73,74,75,76,77,78,79,80,81,82,83,84,85] (Figure 4).

### 3.4. Summary of Findings

**Figure 5:**
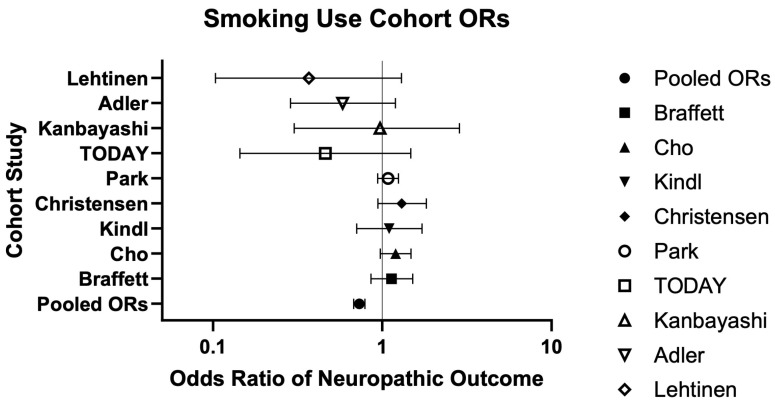
Forest plot of odds ratios for presence of neuropathy/NP according to smoking exposure, as defined by each cohort study. Included studies: [25,26,28,29,30,32,33,34,37].

Quantitative and qualitative synthesis of NP incidence, prevalence, and association with smoking status is available in Table 1 encompassing study characteristics and Table 2, which provides a summary of findings. Of the ten cohort studies assessing smoking consumption and neuropathy/NP, six (60%) reported a significant positive association (*p* < 0.05) [27,28,29,30,32,34], either with increased odds of neuropathy, greater pain severity, or higher neuropathy prevalence (in retrospective cohorts) [27,32] (Table 1). Three studies (30%) found no significant association [26,33,36], and one study (10%) reported significantly lower odds of neuropathy among smokers [25] (Table 1). Collectively, across the ten cohort studies reporting smoking consumption and neuropathy, nine (90%) provided sufficient data for pooled analysis. Among these, the pooled odds ratio was 0.73 [0.68–0.79], and the pooled relative risk was 0.89 [0.86–0.92] [25,26,28,29,30,32,33,34,36] (Table 2 and Figure 5).

The remaining cohort studies each focused on a single outcome, including smoking dependency, lifetime smoking, or smoking cessation. Smoking dependency was significantly associated with increased odds of neuropathy (*p* < 0.05) [31], while lifetime smoking was not significant in multivariate analysis [35], and smoking cessation was significantly associated with a reduction in neuropathy at one year (*p* < 0.05) [37] (Table 1). Among these, the calculated odds ratios were 1.57 [1.45–1.70], 0.68 [0.52–0.89], and 1.04 [0.51–2.16], and the calculated relative risks were 1.20 [1.16–1.23], 0.83 [0.73–0.94], and 1.01 [0.76–1.34], respectively (Table 2).

**Figure 6:**
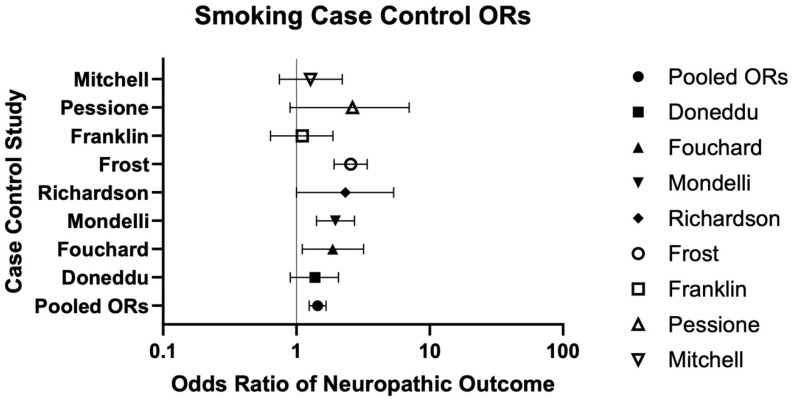
Forest plot of odds ratios for presence of neuropathy/NP according to smoking exposure, as defined by each case–control study. Included studies: [38,39,40,41,42,43,44,45].

A total of nine case–control studies assessed smoking consumption and neuropathy/NP [38,39,40,41,42,43,44,45,46]. Five (56%) reported a statistically significant positive association, including higher smoking prevalence among neuropathy cases, greater neuropathy severity in smokers, and stronger associations with increased pack-years (*p* < 0.05) [40,42,43,44,46] (Table 1). The remaining four studies (44%) found no significant association [38,39,41,45] (Table 1). Among these, eight case–control studies (89%) provided sufficient data to calculate a pooled odds ratio of 1.44 [1.25–1.67] [39,40,41,42,43,44,45,46] (Table 2 and Figure 6).

**Figure 7:**
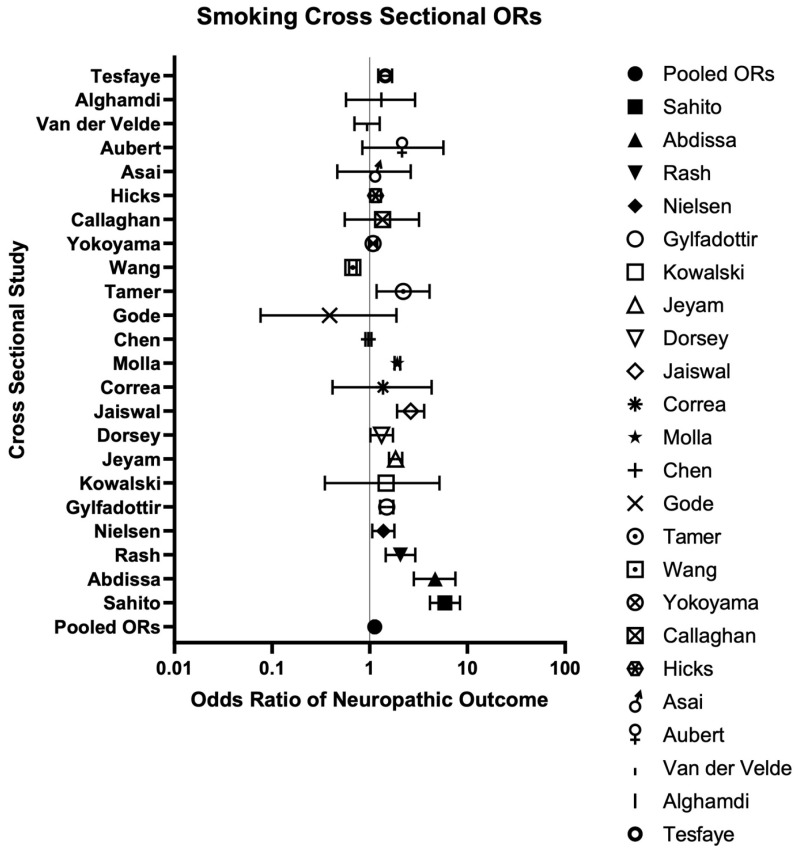
Forest plot of odds ratios for presence of neuropathy/NP according to smoking exposure, as defined by each cross-sectional study. Included studies: [47,48,49,50,52,54,56,57,59,61,62,63,64,65,70,73,75,77,79,80,82,83,85].

Nearly all cross-sectional studies (38/39; 97%) reported smoking consumption in relation to neuropathy/NP, 24 (63%) of which described a statistically significant positive association [47,53,58,60,61,62,63,64,65,66,67,69,70,72,73,74,75,76,77,79,80,82,83,85]. Smoking was significantly associated with a higher prevalence of neuropathy, greater neuropathy severity, and impaired nerve function (Table 1). Smoking history and current smoking status were linked to increased prevalence of neuropathy and NP (*p* < 0.05) [47,58,61,62,63,64,66,67,69,70,74,79,80,82,83,85], with stronger associations observed at higher pack-years (*p* < 0.05) [58,63,66,67,70,74,79,82] (Table 1). Smoking was also significantly associated with electrophysiological abnormalities, including decreased nerve conduction velocity, sensory nerve dysfunction, and autonomic neuropathy (*p* < 0.05) [58,73,76,82] (Table 1). Additionally, smoking severity, addiction level, and duration were significantly higher in individuals with neuropathy (*p* < 0.05) [53,60,65,72,75,77] (Table 1). The remaining 13 studies (33%) did not identify a statistically significant relationship [48,49,50,51,52,54,55,56,57,59,71,78,81], while a single study (3%) reported that never smoking was more frequently observed in individuals with neuropathy than in smokers (~60% vs. ~20%) [68] (Table 1). Cumulatively, 23 studies (59%) provided sufficient data to calculate a pooled odds ratio of 1.12 [1.09–1.15] [47,48,49,50,52,54,56,57,59,61,62,63,64,65,70,73,75,77,79,80,82,83,85] (Table 2 and Figure 7).

The final cross-sectional study, which assessed smoking habits, including frequency, cigarettes per day, and quit attempts, did not report a significant association with neuropathy, but provided sufficient data to calculate an odds ratio of 1.13 [0.50–2.64] [84] (Table 1 and Table 2). Lastly, one case series reported a subjective improvement of NP during smoking abstinence in two participants recovering from surgery for spinal cord injury, with pain recurring upon returning to smoking [86] (Table 1).

## 4. Discussion

This systematic review identified a possible association between smoking and the presence of neuropathy and/or NP. Of the 61 included studies, 35 (57%) reported that current and/or former smoking significantly increased the incidence, prevalence, and/or severity of neuropathic conditions (CIDP, CRPS, DSN, PDN, PN, PoN, SFN, UNE) across various causative etiologies, including diabetes, cancer, and trauma (*p* < 0.05) [27,28,29,30,31,32,34,40,42,43,44,46,47,50,53,58,60,61,63,64,65,66,67,69,70,72,73,74,75,76,77,79,80,82,85]. Comparatively, 21 studies (34%) did not identify a significant relationship [26,33,36,37,38,39,41,45,48,49,51,52,54,55,56,57,59,71,78,81,84], while only 5 (8%) reported a negative association (*p* < 0.05) [25,35,62,68,83]. The overall certainty of evidence was very low due to the observational nature of the studies and a serious risk of bias, primarily due to the inability to objectively measure smoking status (Figure 2, Figure 3 and Figure 4, Table 2). However, despite these limitations, as well as considerable methodological heterogeneity in smoking quantification, neuropathic conditions, and etiological factors, a statistically significant positive association was still observed in the pooled analyses (Table 2). These associations also align with proposed mechanisms by which smoking may exacerbate neuropathic symptoms, including inflammation, neural damage, and dysregulation of pain pathways. Clinically supported mechanisms include altered receptor sensitivity, reduced endogenous opioid activity, and disruption of the hypothalamic–pituitary–adrenal axis. These factors may help explain the increased burden of neuropathy/NP observed among smoking-exposed populations.

### 4.1. Cohort Studies

The estimated effect of smoking status on neuropathy/NP within the included cohort studies was variable and dependent on the degree of exposure. Individuals with tobacco use disorder, who were dependent on smoking, had 57% higher odds of developing diabetic neuropathy compared to non-smokers (1.57 [1.45–1.70]) [31]. Although indirectness and imprecision were not identified as major concerns, the effect estimate is still entirely derived from a single study. As such, generalizability is limited, and the overall certainty of evidence remains low. Therefore, these findings should be interpreted with caution and confirmed by future research (Table 2).

Among cohort studies reporting any degree of smoking exposure, including current and former smoking, effect estimates widely varied. Individuals with any smoking history had 27% lower odds of developing neuropathy/NP compared to non-smokers (0.73 [0.68–0.79]) (Table 2). However, six studies reported increased odds of neuropathy or greater pain severity (*p* < 0.05) [27,28,29,30,32,34], while three found no significant association [26,33,36] (Table 1). Only one study reported a protective effect, which the authors attributed to protopathic bias (*p* = 0.007) [25]. In this case, neuropathy rates may have been inflated in the non-smoking group, as individuals with severe illness from smoking were more likely to have recently quit, underestimating its long-term effects. A similar trend was also identified in a single study assessing lifetime smoking, arbitrarily defined as having smoked >100 cigarettes [35]. The pooled estimate suggests that individuals exceeding this threshold had 37% lower odds of developing chemotherapy-induced peripheral neuropathy (CIPN) compared to those who smoked fewer than 100 cigarettes (Table 2). However, despite this significant association, lifetime smoking was not independently associated with CIPN in multivariate analysis, as reported by the original authors (1.20 [0.87–1.65]), *p* = 0.27) [35] (Table 1). For both smoking status and lifetime smoking, risk of bias and certainty of evidence were consistent across all pooled outcomes, attributable to known limitations (Figure 2, Table 2). Collectively, these factors, including protopathic bias and insufficient confounding adjustment, may explain the observed inverse association in the pooled analyses for both “any” smoking exposure and lifetime smoking.

Lastly, the remaining cohort study assessing smoking cessation reported a significant association with a reduction in PN at one year (*p* < 0.05) [37]. The calculated effects estimate (1.04 [0.51–2.15]) remained insignificant, likely influenced by unadjusted confounders. Additionally, high risk of bias and imprecision further limit its generalizability to a larger population (Figure 2, Table 2). More broadly, high attrition rates, self-report bias, and interventional heterogeneity, remain key challenges in smoking cessation-focused research, reinforcing the need for more robust designs to accurately estimate this effect.

### 4.2. Case–Control Studies

Case–control studies demonstrated a robust positive association between any smoking exposure and neuropathy/NP. The pooled estimate indicates that cases, or those who endorse smoking, have 44% higher odds of neuropathic outcomes compared to controls (1.44 [1.25–1.67]) (Table 2). This effect estimate aligns with published findings demonstrating higher smoking prevalence among neuropathy cases, greater neuropathy severity in smokers, and stronger associations with increased pack-years (*p* < 0.05) [40,42,43,44,46] (Table 1). Overall, most studies identified a positive association, while none reported an inverse relationship. Of the articles that did not find a significant association, smoking status was not the primary endpoint, potentially introducing methodological limitations that may have obscured the true effect [38,39,41,45]. Notably, case–control studies exhibited a lower risk of bias and higher certainty of evidence compared to cohort studies (Figure 3, Table 2). By design, case–control studies also more effectively estimate the impact of smoking exposure on neuropathy/NP development, rather than just prevalence rates. Therefore, case–control studies provided the most robust evidence in this systematic review that any level of smoking exposure significantly increases the odds of developing neuropathy/NP across diverse causative etiologies.

### 4.3. Cross-Sectional Studies

The pooled estimate for cross-sectional studies reporting smoking prevalence among those with neuropathy/NP was 1.12 [1.09–1.15], indicating a significant positive association. Individuals who smoke have 12% higher odds of neuropathic conditions compared to non-smokers (Table 2). This aligns with findings from 23 included studies, which consistently reported a significant relationship between smoking and neuropathy/NP. Specifically, current and former smoking, daily smoking, and smoking history were associated with increased odds of prevalent neuropathy (*p* < 0.05) [47,50,53,58,60,61,63,64,65,66,67,69,70,72,73,74,75,76,77,79,80,82,85], greater pain severity (*p* < 0.05) [50,53,65], and worse nerve function (*p* < 0.05) [58,77,79,82] (Table 1). Of the remaining cross-sectional studies, 12 did not report significant prevalence rates [48,49,51,52,54,55,56,57,59,71,78,81], while 3 studies demonstrated an inverse effect, where smoking was associated with decreased odds of prevalent neuropathy/NP [62,68,83]. In these studies, smoking was a secondary demographic variable rather than a primary outcome, and robust multivariate analyses were not reported, leaving findings susceptible to significant confounding from external lifestyle factors, including diet, exercise, and alcohol consumption. Therefore, the underlying effects of smoking on neuropathy/NP prevalence may not have been reliably estimated in the three studies demonstrating an inverse relationship between smoking status and neuropathy/NP (Table 1). Otherwise, cross-sectional studies had the lowest risk of bias among all study designs, only limited by a reliance on self-reported smoking status. Likewise, considerable variability in effect estimates, likely due to methodological differences, contributed to inconsistencies across studies, while the secondary reporting of smoking exposure contributed to a lesser extent (Figure 4, Table 2).

The remaining cross-sectional study specifically assessed the impact of lifetime smoking on neuropathy/NP prevalence, resulting in a calculated effects estimate of 1.13 [0.50–2.64], indicating no significant association. The overall risk of bias was very serious, and certainty of evidence remained very low; however, additional studies examining this metric are necessary to better quantify this relationship [84] (Figure 4, Table 1 and Table 2). Lastly, a single case series reported subjective improvement in NP during smoking abstinence, with symptoms returning upon resumption of smoking, aligning with the broader trend identified in this systematic review [86] (Table 1). Ultimately, pooled estimates and findings from cross-sectional studies in this review support a positive correlation between smoking status and neuropathy/NP prevalence, reinforcing the association observed across study designs.

### 4.4. Limitations

The limitations identified within this systematic review are largely driven by clinical and methodological heterogeneity present within the included body of literature. Given the pervasive nature of neuropathy/NP, the included studies encompassed a range of underlying causative etiologies, ultimately impacting interpretability. Likewise, as previously described, significant inconsistencies in the classification and quantification of smoking exposure likely introduced bias, reducing the overall certainty of evidence and comparability across studies. These discrepancies may have also contributed to protopathic biases, whereby early neuropathic symptoms influenced smoking behavior, obscuring directionality and limiting causal inference. Ultimately, the overall risk of bias was substantially impacted by an inability to objectively measure smoking status, as most studies relied on subjective self-reports, lacking physiological confirmation. No studies reported biometric or physiologic corroboration of smoking exposure or status. Additionally, due to a paucity of data, some outcome estimates including lifetime smoking and smoking cessation were based on a single study, resulting in imprecision. Given the nature of addiction, and propensity for relapse, smoking cessation is particularly difficult to reliably measure, particularly when outcomes are measured temporally close to reported cessation. Likewise, weight gain following cessation may confound diabetes- and impact/compressive-related neuropathy outcomes, complicating interpretability. More robust studies with granular biological correlates are needed to better understand this impact. Lastly, lifestyle parameters such as smoking status were often recorded as secondary outcomes in the broader literature, reported for covariate adjustment rather than exposures of interest in primary analyses. This contributed to greater indirectness and limited the generalizability of findings. Despite these limitations, pooled analyses demonstrated a consistent and statistically significant positive relationship between smoking exposure and neuropathy/NP incidence, prevalence, and severity.

## 5. Conclusions

This systematic review identified a wide breadth of literature summarizing the impact of smoking status on neuropathy/NP incidence, prevalence, and severity, due to various etiologies. The reported data and calculated pooled effects estimates suggests that smoking may be associated with greater pain severity and neuropathy/NP prevalence, while cessation reduces and dependency increases the risk of incident cases. Overall, smoking status plays a highly significant role in the pathogenesis, morbidity, and burden of very common, non-infectious diseases, such as diabetes, cancers, and trauma. This systematic review also revealed a notable gap in the literature, as eligible studies on infectious etiologies, in particular, leprosy, were markedly lacking despite their continued global impact. From an infectious disease standpoint, leprosy remains a major cause of chronic peripheral NP and is likely exacerbated by smoking, yet remains underrepresented in intervention-based research. This observational evidence suggests that smoking may compound neuropathic symptoms in leprosy via delayed wound healing, increased ulceration, and heightened inflammatory responses. These potential interactions, although less frequently reported, underscore the importance of investigating lifestyle risk factors beyond well-studied non-infectious frameworks. As such, patients with leprosy may benefit from additional counseling regarding smoking-related risks and symptom management, which should be integrated into existing clinical guidelines and safety protocols [87]. In summary, smoking significantly exacerbates neuropathy/NP, and quitting may serve as a low-risk, low-cost, low-tech, and potentially durable adjunctive therapy, particularly in those with common non-infectious conditions. While pooled effects were broadly aligned, the strength of inference remains limited by methodological variability and the lack of objective exposure assessment. Moving forward, large-scale, comprehensive cessation interventional trials are warranted to strengthen the current body of evidence for less common infectious diseases, such as leprosy, and to support recovery efforts in at-risk populations where it remains endemic.

## Data Availability

The raw data supporting the conclusions of this article will be made available by the authors on request.

## Abbreviations

The following abbreviations are used in this manuscript:

BMI	Body mass index
Ca	Cases
CIDP	Chronic inflammatory demyelinating polyradiculoneuropathy
CIPN	Chemotherapy-induced peripheral neuropathy
CIPN20	European Organization for Research and Treatment of Cancer 20-item scale
CMAP	Compound muscle action potential
Co	Controls
COPD	Chronic obstructive pulmonary disease
CP	Chronic pain
CRPS	Complex regional pain syndrome
CTCAE	National Cancer Institute Common Terminology Criteria for Adverse Events
CV	Conduction velocity
DM	Diabetes mellitus
DN	Diabetic neuropathy
DPN	Diabetic peripheral neuropathy
DPoN	Diabetic polyneuropathy
DPoNS	Diabetic polyneuropathy-related sensory symptoms/signs
DSN	Distal symmetric neuropathy
DSPoN	Distal sensory polyneuropathy
DSyPN	Distal symmetrical peripheral neuropathy
GABA	Gamma-aminobutyric acid
IDDM	Insulin-dependent diabetes mellitus
IEFND	Intraepidermal nerve fiber density
KSA	Kingdom of Saudi Arabia
LANSS	Leeds assessment of neuropathic symptoms and signs
LED	Lower-extremity disease
LLBP	Localized lower-back pain
MNSI	Michigan neuropathy screening instrument
MSK	Musculoskeletal pain
NCV	Nerve conduction velocity
NDS	Neuropathy disability score
ND	Neurophysiologically deteriorated
NIDDM	Non-insulin-dependent diabetes mellitus
NNT	Number needed to treat
NP	Neuropathic pain
NSRC	Non-standardized regression coefficient
PDN	Painful diabetic neuropathy
PDPN	Painful diabetic peripheral neuropathy
PN	Peripheral neuropathy
PNBP	Peripheral neuropathic back pain
PNQ	Patient neurotoxicity questionnaire
PoN	Polyneuropathy
PSUD	Polysubstance use disorder group (tobacco, alcohol, +1 other)
PY	Person-years
SCI	Spinal cord injury
SFN	Small-fiber neuropathy
SNAPA	Sensory nerve action potential amplitude
SRC	Standardized regression coefficient
T1DM	Type 1 diabetes mellitus
T2DM	Type 2 diabetes mellitus
TAUD	Tobacco and alcohol use disorder group
TUD	Tobacco use disorder group
UDPoN	Unknown-status diabetic polyneuropathy
UN	Ulnar neuropathy
UNE	Ulnar neuropathy of the elbow
VPT	Vibration perception threshold
WP	Widespread pain
